# Predictive factors and pharmacological preventive interventions for atrial fibrillation after aortic valve replacement

**DOI:** 10.1186/s13019-025-03577-6

**Published:** 2025-08-09

**Authors:** Lu Chen, Yu Liu, Junmei Ge

**Affiliations:** Department of Cardiovascular Medicine, Zhongda Hospital Affiliated to Southeast University, Nanjing, 210009 Jiangsu China

**Keywords:** Postoperative atrial fibrillation after aortic valve replacement, Predictive factors, Atorvastatin, Metoprolol, Pharmacological prevention

## Abstract

**Objective:**

This study aims to investigate the predictive factors for postoperative atrial fibrillation (POAF) following aortic valve replacement (AVR) and evaluate the preventive effect of combined atorvastatin and metoprolol therapy on POAF.

**Methods:**

This study employed a mixed design of retrospective cohort analysis and prospective randomized controlled trial, including 268 patients who underwent isolated AVR from January 1, 2022, to March 31, 2024. The 168 patients from January 1, 2022, to May 31, 2023, were analyzed for POAF predictive factors, while 100 patients from June 1, 2023, were included in the prospective trial. The intervention group (*n* = 50) received combined atorvastatin and metoprolol treatment starting 7 days before surgery.

**Results:**

Multivariate logistic regression analysis identified age (OR = 1.12, 95% CI: 1.04–1.20, *p* = 0.003), history of stroke (OR = 10.94, 95% CI: 1.32–90.66, *p* = 0.027), EuroSCORE II (OR = 2.90, 95% CI: 1.61–5.20, *p* < 0.001), NT-proBNP level (OR = 1.002, 95% CI: 1.001–1.004, *p* = 0.009), hs-CRP level (OR = 1.55, 95% CI: 1.16–2.07, *p* = 0.003), and operation time (OR = 1.02, 95% CI: 1.01–1.04, *p* = 0.008) as independent predictors of POAF. Pharmacological intervention significantly reduced POAF incidence (*p* = 0.005) and shortened hospital stay (*p* < 0.001), ICU stay (*p* = 0.002), and mechanical ventilation time (*p* < 0.001). The AUC of the predictive model was 0.952, with a calibrated C-statistic of 0.904. Decision curve analysis demonstrated significant clinical utility across multiple risk thresholds.

**Conclusion:**

Age, history of stroke, EuroSCORE II, NT-proBNP and hs-CRP levels, and operation time are independent predictors of POAF. Combined preventive treatment with atorvastatin and metoprolol reduced POAF incidence and postoperative hospital stay, showing promising clinical application prospects.

Surgical aortic valve replacement (SAVR) is an effective treatment for severe aortic valve disease, significantly improving patient symptoms and prognosis [1]. Postoperative atrial fibrillation (POAF), as one of the most common complications following SAVR, seriously affects patient recovery and long-term prognosis [2]. Previous studies have reported that the incidence of POAF after SAVR ranges from 11.1 to 84% [3], not only prolonging hospital stay and increasing medical costs but also closely associated with long-term adverse events such as stroke and cognitive decline [4, 5].

The pathogenesis of POAF is complex, involving multiple pathophysiological processes including inflammatory response, oxidative stress, autonomic dysfunction, and atrial remodeling [6]. Surgery-related factors (such as surgical trauma, ischemia-reperfusion injury) and patient-intrinsic factors (such as age, comorbidities) interact to lead to the occurrence of POAF [7, 8]. However, there is currently a lack of high-performance POAF prediction models specifically for SAVR patients. While several studies have developed prediction models for POAF following cardiac surgery, most focus on coronary artery bypass grafting or mixed cardiac procedures, with limited data specifically addressing isolated aortic valve replacement. Furthermore, existing models often lack external validation and demonstrate inconsistent discriminative performance across different populations.

Given the high incidence and adverse effects of POAF, preventive measures are crucial. Currently, pharmacological prevention is one of the most commonly used strategies. β-blockers are widely used in POAF prevention due to their ability to inhibit sympathetic activity [9]. Among β-blockers, both metoprolol and carvedilol have shown efficacy in POAF prevention. While recent meta-analyses suggest potential superiority of carvedilol over metoprolol in reducing POAF incidence, metoprolol remains the most widely studied and clinically established β-blocker for perioperative use. Our choice of metoprolol was based on its extensive evidence base in cardiac surgery patients, favorable safety profile, and established perioperative protocols at our institution. Additionally, metoprolol’s shorter half-life provides better perioperative hemodynamic control, which is particularly important in patients undergoing valve surgery. Statins, with their pleiotropic effects (anti-inflammatory, antioxidant, improving endothelial function, etc.), have also shown preventive efficacy [10]. Recent studies suggest that combination therapy may produce synergistic effects, enhancing preventive efficacy [11, 12]. However, high-quality randomized controlled trials targeting SAVR patients are still lacking.

Current prediction models for POAF demonstrate significant heterogeneity in terms of included variables, model performance, and clinical applicability. Many existing models incorporate numerous variables that may not be readily available in clinical practice, limiting their utility. Moreover, most studies have focused on single-center experiences without adequate external validation, raising questions about generalizability across different surgical centers and patient populations.

Based on the current research status, this study aims to comprehensively evaluate potential predictive factors for POAF after SAVR, including clinical characteristics, surgery-related factors, and biomarkers; construct and validate a high-performance POAF risk prediction model that is both accurate and clinically practical; and assess the efficacy and safety of combined atorvastatin and metoprolol preventive therapy in SAVR patients. This research addresses the critical gap in evidence-based POAF prevention strategies specifically for isolated aortic valve replacement procedures.

## Materials and methods

### Study design and overview

This study employed a two-phase mixed design to investigate predictive factors for postoperative atrial fibrillation (POAF) following aortic valve replacement and evaluate pharmacological preventive interventions. The study was conducted as follows: Phase I (January 1, 2022, to May 31, 2023) consisted of a retrospective cohort study to identify POAF predictive factors and develop a prediction model; Phase II (June 1, 2023, to March 31, 2024) was a prospective randomized controlled trial to evaluate the efficacy of combined atorvastatin and metoprolol prophylaxis. This design allows for robust predictor identification from the larger retrospective cohort while providing high-quality evidence for intervention efficacy through the prospective trial.

The study population comprised consecutive adult patients who underwent isolated surgical aortic valve replacement (SAVR) at our hospital between January 1, 2022, and March 31, 2024. A total of 268 patients were included, with 168 patients from Phase I used for retrospective analysis, and 100 patients from Phase II enrolled in the prospective randomized controlled trial. The overall study population included 152 males and 116 females. Patient age ranged from 45 to 78 years, with a mean age of 63.2 ± 9.5 years. Patients were divided into POAF and non-POAF groups based on the occurrence of postoperative atrial fibrillation. POAF was defined as newly onset atrial fibrillation occurring within 30 days postoperatively, lasting more than 5 min or requiring pharmacological/electrical cardioversion.

The prospective randomized controlled trial (Phase II) was registered at ClinicalTrials.gov (Registration Number: ) and received separate ethical approval from our hospital’s Medical Ethics Committee (Approval No.: ) in addition to the overall study approval.

Inclusion criteria: (1) Age ≥ 18 years; (2) Undergoing isolated SAVR for aortic valve disease; (3) Preoperative sinus rhythm without history of sustained arrhythmias; (4) No cardiac surgery or intervention within 3 months before the operation; (5) Complete clinical data, including preoperative assessment, surgical records, and postoperative monitoring data; (6) Patient or legal representative consent to participate and signed informed consent.

Exclusion criteria: (1) Preoperative diagnosis of paroxysmal, persistent, or permanent atrial fibrillation; (2) Concomitant cardiac lesions requiring surgical intervention (e.g., coronary artery disease, mitral valve disease); (3) Previous cardiac surgery history; (4) Severe hepatic or renal dysfunction (Child-Pugh class C or eGFR < 30 mL/min/1.73 m²); (5) Hyperthyroidism or hypothyroidism; (6) Use of antiarrhythmic drugs within 3 months preoperatively; (7) Malignant tumors or life expectancy less than 1 year; (8) Pregnant or lactating women; (9) Patients with mental illness or unable to comply with follow-up; (10) Participation in other clinical trials that may affect the judgment of this study’s results.

This study protocol was approved by our hospital’s Medical Ethics Committee (Approval No.: CARDIAC-2022-0115), and all patients or their legal representatives signed informed consent forms.

### Phase I: retrospective cohort study (Predictive factor analysis)

#### Study population and data collection

The retrospective phase included 168 consecutive patients who underwent isolated SAVR from January 1, 2022, to May 31, 2023. The primary objective of this phase was to identify independent predictors of POAF and develop a clinically applicable prediction model. All patients met the inclusion and exclusion criteria outlined above.

Standardized electronic case report forms were used to collect the following data: (1) Baseline characteristics: age, gender, body mass index (BMI), medical history, preoperative medications, etc.; (2) Preoperative assessment: NYHA functional class, EuroSCORE II, laboratory test results (including NT-proBNP and hs-CRP), echocardiographic parameters, ECG parameters, etc.; (3) Surgery-related data: operation time, cardiopulmonary bypass time, aortic cross-clamp time, etc.; (4) Postoperative monitoring data: cardiac rhythm, hemodynamic parameters, complications, etc. All data were managed using REDCap software.

### Phase II: prospective randomized controlled trial

#### Sample size calculation

Sample size calculation was performed using PASS 15.0 software based on preliminary data from our institution. Assuming a baseline POAF incidence of 35% in the control group and expecting a 50% relative reduction (17.5% absolute incidence) in the intervention group, with α = 0.05 (two-tailed) and β = 0.20 (power = 80%), the calculated sample size was 82 patients (41 per group). Accounting for a 20% dropout rate, we planned to enroll 100 patients (50 per group). This sample size provides adequate power to detect clinically meaningful differences in POAF incidence between groups.

#### Randomization and blinding

In the prospective randomized controlled trial phase, 100 patients were randomized 1:1 into intervention (*n* = 50) and control (*n* = 50) groups using computer-generated block randomization with varying block sizes (4, 6, 8) to ensure allocation concealment. The randomization sequence was generated by an independent statistician and kept in sealed opaque envelopes. Stratified randomization was not performed due to the relatively small sample size and the risk of creating imbalanced groups within strata. However, baseline characteristics were carefully monitored and compared between groups to ensure comparability. Researchers and patients were not blinded to group allocation due to the nature of the intervention, but outcome assessors remained blinded to treatment allocation when evaluating POAF occurrence and other endpoints.

#### Intervention protocol

The 7-day preoperative intervention period was selected based on previous pharmacokinetic studies demonstrating that atorvastatin requires 5–7 days to achieve steady-state concentrations and maximal pleiotropic effects, while metoprolol reaches therapeutic β-blockade within 24–48 h. The combination allows for optimal anti-inflammatory and cardioprotective effects before surgical stress.

The intervention group received combined treatment with atorvastatin (Lipitor, Pfizer, 40 mg once daily) and metoprolol tartrate (Betaloc, AstraZeneca, 25 mg twice daily) starting 7 days preoperatively and continuing until discharge. The control group received routine preoperative preparation without additional use of these medications. Baseline characteristics including age, gender, EuroSCORE II, NYHA class, and comorbidities were compared between intervention and control groups to ensure comparability. Other perioperative management strategies remained consistent between groups.

### Surgical procedure and perioperative management

All surgeries were performed by experienced cardiac surgeons using standard median sternotomy and cardiopulmonary bypass techniques (Stöckert S5 heart-lung machine, Germany). Mechanical valves (On-X, CryoLife, USA) or bioprosthetic valves (Trifecta GT, Abbott, USA) were selected based on patient conditions. Intraoperative monitoring included Swan-Ganz catheter and transesophageal echocardiography (Vivid E95, GE Healthcare, USA) for hemodynamic monitoring and valve function assessment. Postoperatively, patients were transferred to the cardiac surgery intensive care unit for continuous cardiac and hemodynamic monitoring (IntelliVue MX800, Philips, Netherlands) for at least 7 days. Vital signs were recorded every 4 h, and 12-lead ECG (MAC 5500 HD, GE Healthcare, USA) was performed daily. Transthoracic echocardiography (Vivid E95, GE Healthcare, USA) was routinely performed on postoperative days 1, 3, 5, 7, and before discharge.

### Outcome measures and definitions

The primary endpoint was the occurrence of POAF, defined as newly onset atrial fibrillation occurring within 30 days postoperatively, lasting more than 30 min or requiring pharmacological/electrical cardioversion. POAF diagnosis was based on continuous ECG monitoring and daily 12-lead ECG examinations, with all suspected atrial fibrillation events independently reviewed and confirmed by two experienced cardiologists. Secondary endpoints included: (1) Length of hospital stay; (2) ICU length of stay; (3) Duration of mechanical ventilation; (4) Major adverse cardiovascular events (MACE, including cardiac death, non-fatal myocardial infarction, non-fatal stroke, and unplanned revascularization); (5) 30-day readmission rate; (6) Postoperative bleeding volume; (7) Postoperative transfusion requirements; (8) Postoperative renal dysfunction (defined as serum creatinine increase > 0.3 mg/dL or > 50% from baseline); (9) 30-day all-cause mortality; (10) Drug-related adverse reactions.

### Statistical methods

Statistical analyses were performed separately for the retrospective cohort study (Phase I) and prospective randomized controlled trial (Phase II) to avoid analytical bias from mixing different study designs. All statistical tests were two-sided, with *P* < 0.05 considered statistically significant.

#### Phase I statistical analysis (Retrospective cohort)

Statistical analyses were performed using SPSS 26.0 software (IBM Corp., Armonk, NY, USA) and R software version 4.1.0. The normality of continuous variables was confirmed using the Shapiro-Wilk test. Normally distributed continuous variables were expressed as mean ± standard deviation (Mean ± SD) and compared using independent samples t-test. Non-normally distributed continuous variables were expressed as median (interquartile range) [M(IQR)] and compared using the Mann-Whitney U test. Categorical variables were expressed as frequency (percentage) [n(%)] and compared using chi-square test or Fisher’s exact test.

**Fig. 1:**
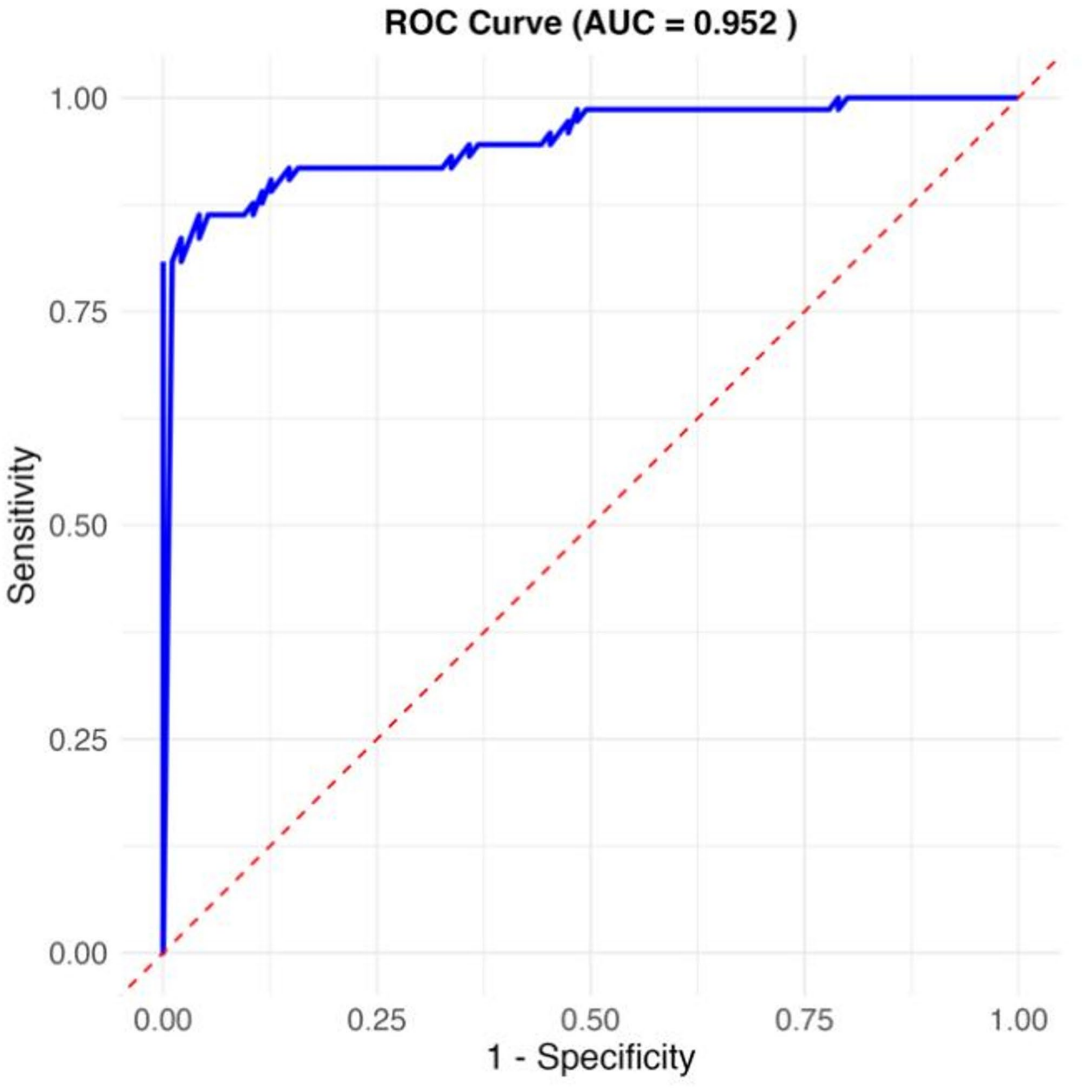
ROC curve

To identify independent predictors of postoperative atrial fibrillation (POAF), univariate logistic regression analysis was first performed. Variables with *P* < 0.10 in the univariate analysis were included in the multivariate logistic regression model, and Akaike Information Criterion (AIC) was used for model selection to identify the optimal combination of predictors while avoiding overfitting. The model’s discriminative ability was assessed using the area under the receiver operating characteristic (ROC) curve (AUC), and calibration was evaluated using the Hosmer-Lemeshow test Fig. 1.

Based on the results of the multivariate logistic regression model, a simplified Nomogram prediction model containing 3–4 key variables was constructed to enhance clinical practicality. Internal validation of the model was performed using 1000 bootstrap resamples to calculate the corrected C-statistic. Additionally, a calibration curve was plotted to assess the consistency between predicted probabilities and actual observed results, and decision curve analysis (DCA) was used to evaluate the clinical utility of the model.

#### Phase II statistical analysis (Randomized controlled trial)

For the prospective RCT phase, baseline characteristics between intervention and control groups were compared using appropriate statistical tests to confirm successful randomization. The primary endpoint (POAF incidence) was analyzed using chi-square test or Fisher’s exact test. Secondary continuous endpoints were analyzed using independent samples t-test or Mann-Whitney U test as appropriate. Time-to-event analyses were performed using Kaplan-Meier survival analysis with log-rank tests. Both intention-to-treat and per-protocol analyses were planned, with the intention-to-treat analysis serving as the primary analysis.

Safety analysis included all patients who received at least one dose of study medication, with adverse events categorized by severity and relationship to study drugs.

Patients were followed up at outpatient clinics at 1 month and 3 months post-discharge, and every 6 months thereafter. Each follow-up included symptom assessment, physical examination, 12-lead ECG, transthoracic echocardiography, and laboratory tests.

## Results

This study included a total of 268 patients who underwent isolated aortic valve replacement, with 168 patients in Phase I (retrospective analysis) and 100 patients in Phase II (prospective randomized controlled trial).

### Phase I: retrospective cohort study results

#### Patient characteristics and POAF incidence

Among the 168 patients in the retrospective analysis, 73 (43.45%) developed postoperative atrial fibrillation (POAF). The baseline characteristics were well-distributed across the cohort, with no significant imbalances that would affect the validity of the predictive model development.

#### Univariate analysis of POAF-related factors

Table 1 presents the results of univariate logistic regression analysis for POAF-related factors. The analysis systematically evaluated patient demographics, comorbidities, cardiac function parameters, biomarkers, and surgical factors. Age was an important risk factor, with the POAF group having a significantly higher mean age (66.55 ± 8.93 years) compared to the non-POAF group (58.86 ± 8.20 years, *p* < 0.001). A history of stroke was more prevalent in the POAF group (16.44%) compared to the non-POAF group (5.26%, *p* = 0.034). Cardiac functional status was also associated with POAF risk, with a higher proportion of NYHA III-IV patients in the POAF group (64.38%) compared to the non-POAF group (36.84%, *p* = 0.005). The EuroSCORE II was significantly higher in the POAF group (3.50 ± 1.42) compared to the non-POAF group (2.20 ± 1.08, *p* < 0.001).

**Table 1:** Univariate logistic regression analysis of POAF-Related factors

Variable	No POAF (*n* = 95)	POAF (*n* = 73)	Test Statistic	*p*-value
Age (years)	58.86 ± 8.20	66.55 ± 8.93	-5.728	< 0.001
Male, n (%)	50 (52.63%)	44 (60.27%)	0.693	0.405
BMI (kg/m²)	25.66 ± 3.03	26.57 ± 3.68	-1.704	0.091
Hypertension, n (%)	54 (56.84%)	48 (65.75%)	1.026	0.311
Diabetes Mellitus, n (%)	29 (30.53%)	30 (41.10%)	1.587	0.208
COPD, n (%)	12 (12.63%)	12 (16.44%)	0.227	0.634
Previous Stroke, n (%)	5 (5.26%)	12 (16.44%)	4.506	0.034
NYHA Class III-IV, n (%)	35 (36.84%)	47 (64.38%)	12.686	0.005
EuroSCORE II	2.20 ± 1.08	3.50 ± 1.42	-6.484	< 0.001
LVEF (%)	56.68 ± 6.99	54.01 ± 8.43	2.186	0.030
LAVI (mL/m²)	33.90 ± 6.16	39.29 ± 7.54	-4.962	< 0.001
Aortic Valve Area (cm²)	0.93 ± 0.20	0.81 ± 0.19	3.841	< 0.001
Mean Transvalvular Gradient (mmHg)	41.90 ± 10.39	48.95 ± 14.41	-3.534	< 0.001
NT-proBNP (pg/mL)	605.20 ± 326.95	968.74 ± 461.77	-5.715	< 0.001
hs-CRP (mg/L)	3.26 ± 1.83	5.04 ± 2.36	-5.327	< 0.001
Surgery Time (min)	177.92 ± 31.63	201.37 ± 33.42	-4.613	< 0.001
CPB Time (min)	91.98 ± 20.36	107.77 ± 21.58	-4.816	< 0.001
Aortic Cross-Clamp Time (min)	70.35 ± 14.37	74.24 ± 14.59	-1.721	0.087

POAF: postoperative atrial fibrillation; BMI: body mass index; COPD: chronic obstructive pulmonary disease; NYHA: New York Heart Association; LVEF: left ventricular ejection fraction; LAVI: left atrial volume index; NT-proBNP: N-terminal pro-brain natriuretic peptide; hs-CRP: high-sensitivity C-reactive protein; CPB: cardiopulmonary bypass

##### Echocardiographic parameters revealed important structural predictors

the POAF group had significantly higher left atrial volume index (LAVI) compared to the non-POAF group (39.29 ± 7.54 vs. 33.90 ± 6.16 mL/m², *p* < 0.001), while aortic valve area was significantly smaller (0.81 ± 0.19 vs. 0.93 ± 0.20 cm², *p* < 0.001). The mean transvalvular pressure gradient was significantly higher in the POAF group (48.95 ± 14.41 mmHg) compared to the non-POAF group (41.90 ± 10.39 mmHg, *p* < 0.001).

##### Biomarker analysis demonstrated the inflammatory and hemodynamic basis of POAF

the POAF group had significantly higher levels of NT-proBNP (968.74 ± 461.77 pg/mL) and hs-CRP (5.04 ± 2.36 mg/L) compared to the non-POAF group (605.20 ± 326.95 pg/mL and 3.26 ± 1.83 mg/L, respectively, both *p* < 0.001).

##### Surgery-related factors showed clear associations with POAF risk

the POAF group had significantly longer operation time (201.37 ± 33.42 min) and cardiopulmonary bypass time (107.77 ± 21.58 min) compared to the non-POAF group (177.92 ± 31.63 min and 91.98 ± 20.36 min, respectively, both *p* < 0.001).

#### Multivariate analysis and independent predictors

Table 2 presents the results of multivariate logistic regression analysis for independent predictors of POAF. Variables with *p* < 0.10 in the univariate analysis were included, and AIC-based model selection was employed to identify the optimal combination of predictors. The final model identified six independent predictors: age (OR = 1.12, 95% CI: 1.04–1.20, *p* = 0.003), history of stroke (OR = 10.94, 95% CI: 1.32–90.66, *p* = 0.027), EuroSCORE II (OR = 2.90, 95% CI: 1.61–5.20, *p* < 0.001), NT-proBNP level (OR = 1.002, 95% CI: 1.001–1.004, *p* = 0.009), hs-CRP level (OR = 1.55, 95% CI: 1.16–2.07, *p* = 0.003), and operation time (OR = 1.02, 95% CI: 1.01–1.04, *p* = 0.008). Although NYHA classification, LAVI, and aortic valve area showed significance in univariate analysis, they did not emerge as independent predictors after adjustment for other factors, indicating potential collinearity with the retained variables.

**Table 2:** Multivariate logistic regression analysis of independent predictors for POAF

Variable	Beta	SE	Wald Chi-Square	OR	CI Lower	CI Upper	*p*-value
Age (years)	0.11	0.04	9.013	1.12	1.04	1.20	0.003
Previous Stroke, n (%)	2.39	1.08	4.916	10.94	1.32	90.66	0.027
NYHA Class II, n (%)	-0.10	0.80	0.016	0.90	0.19	4.32	0.898
NYHA Class III, n (%)	-0.08	0.84	0.008	0.93	0.18	4.84	0.927
NYHA Class IV, n (%)	0.39	0.97	0.158	1.47	0.22	9.88	0.691
EuroSCORE II	1.06	0.30	12.681	2.90	1.61	5.20	< 0.001
LVEF (%)	0.02	0.04	0.280	1.02	0.94	1.11	0.597
LAVI (mL/m²)	0.06	0.04	1.591	1.06	0.97	1.15	0.207
Aortic Valve Area (cm²)	-2.68	1.59	2.847	0.07	0.003	1.54	0.092
Mean Gradient (mmHg)	0.04	0.02	3.250	1.04	1.00	1.08	0.071
NT-proBNP (pg/mL)	0.002	0.001	6.910	1.002	1.001	1.004	0.009
hs-CRP (mg/L)	0.44	0.15	8.711	1.55	1.16	2.07	0.003
Surgery Time (min)	0.02	0.01	7.031	1.02	1.01	1.04	0.008
CPB Time (min)	0.03	0.01	3.318	1.03	1.00	1.06	0.069

POAF: postoperative atrial fibrillation; NYHA: New York Heart Association; LVEF: left ventricular ejection fraction; LAVI: left atrial volume index; NT-proBNP: N-terminal pro-brain natriuretic peptide; hs-CRP: high-sensitivity C-reactive protein; CPB: cardiopulmonary bypass; OR: odds ratio; CI: confidence interval; SE: standard error

#### Prediction model development and validation

**Fig. 2:**
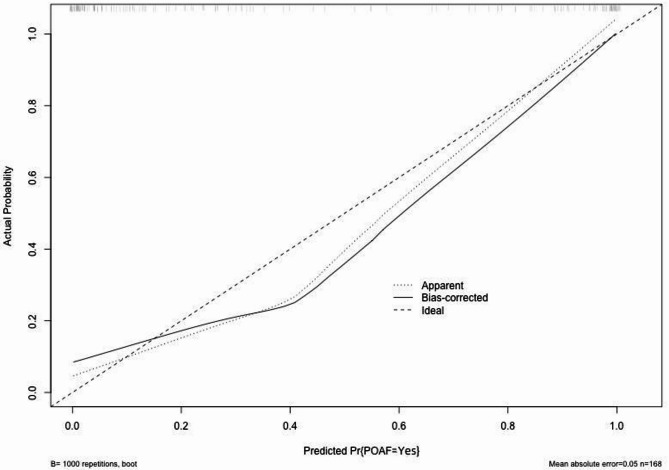
Calibration curve

**Fig. 3:**
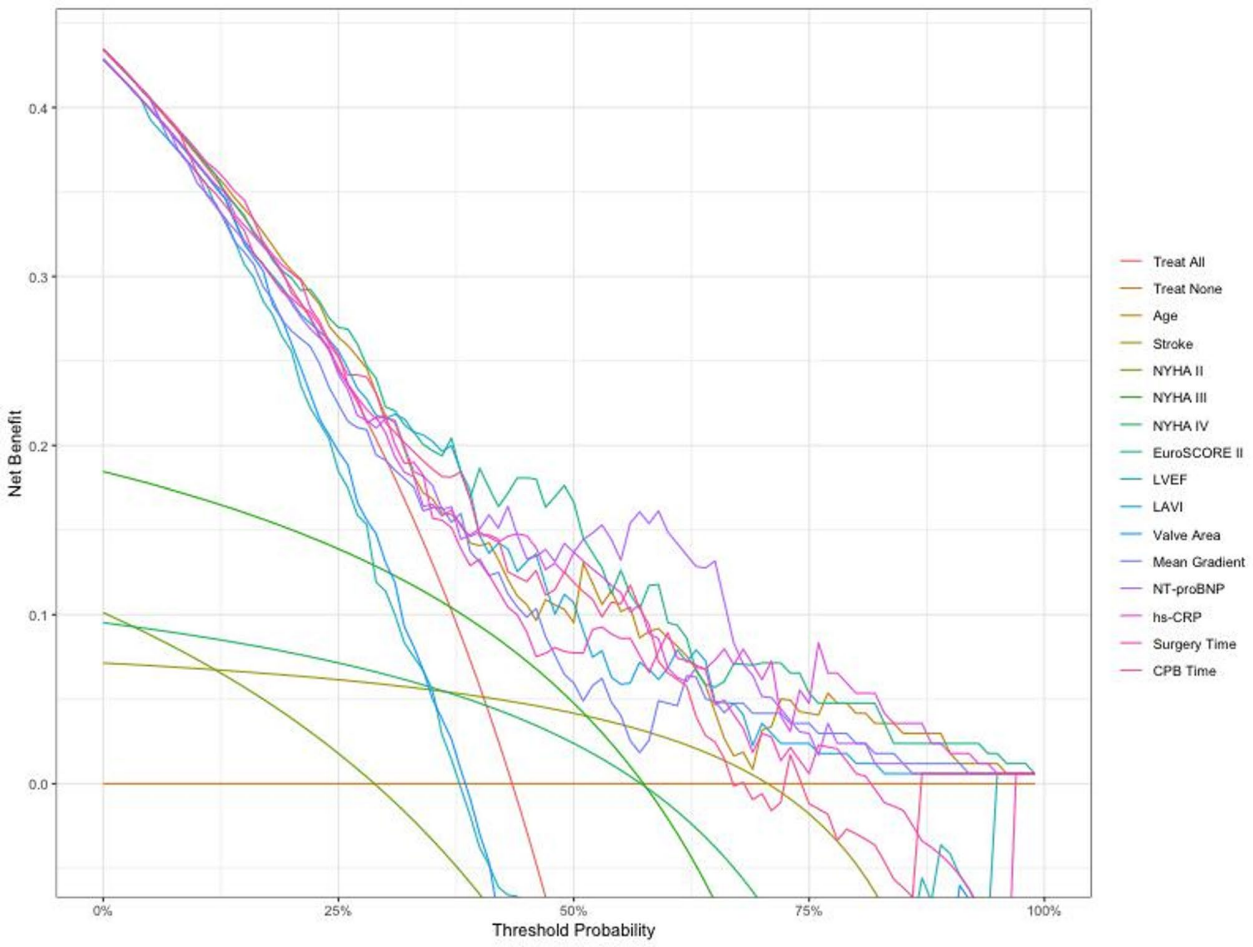
Decision curve analysis (DCA)

**Fig. 4:**
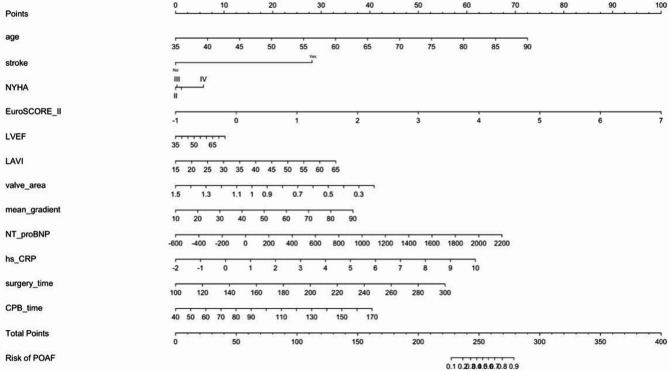
Nomogram prediction model

A simplified nomogram prediction model was constructed incorporating the four most clinically relevant and readily available predictors: age, EuroSCORE II, NT-proBNP, and hs-CRP levels Fig. 1. The model demonstrated excellent discriminative ability (AUC = 0.952, calibrated C-statistic = 0.904) and good calibration (mean absolute error = 0.05) Figs. 2 and 3. Internal validation using 1000 bootstrap resamples confirmed model stability with a corrected C-statistic of 0.922. Decision curve analysis demonstrated significant clinical utility across risk thresholds of 10%-80% Fig. 4, indicating superior performance compared to treat-all or treat-none strategies.

### Phase II: prospective randomized controlled trial results

#### Patient characteristics and randomization success

Table 3a presents baseline characteristics comparison between intervention and control groups. Successful randomization was confirmed with no statistically significant differences in age (64.2 ± 8.1 vs. 65.8 ± 9.3 years, *p* = 0.346), gender distribution (52% vs. 58% male, *p* = 0.549), EuroSCORE II (2.8 ± 1.2 vs. 3.1 ± 1.4, *p* = 0.239), or other major baseline characteristics, ensuring valid comparison between groups.

**Table 3:** Comparison of POAF incidence and clinical outcomes between intervention and control groups

Variable	Intervention (*n* = 50)	Control (*n* = 50)	Test Statistic	*p*-value
Incidence of POAF; n (%)	8 (16.00%)	17 (34.00%)	4.332	0.037
Hospital Stay (days)	7.37 ± 1.84	9.11 ± 2.46	5.194	< 0.001
ICU Stay (hours)	30.12 ± 7.47	35.26 ± 13.31	3.103	0.002
Mechanical Ventilation Time (hours)	10.48 ± 4.72	15.55 ± 6.10	6.037	< 0.001
Major Adverse Cardiovascular Events; n (%)	2 (4.00%)	6 (12.00%)	2.174	0.140
30-day Readmission Rate; n (%)	3 (6.00%)	7 (14.00%)	1.778	0.182
Postoperative Bleeding (mL)	324.69 ± 101.36	343.26 ± 116.50	1.104	0.271
Postoperative Blood Transfusion; n (%)	6 (12.00%)	8 (16.00%)	0.332	0.564
Postoperative Renal Dysfunction; n (%)	3 (6.00%)	7 (14.00%)	1.778	0.182
30-day Mortality; n (%)	1 (2.00%)	2 (4.00%)	0.344	0.557

POAF: postoperative atrial fibrillation; ICU: intensive care unit

#### Primary endpoint: POAF incidence

The primary analysis demonstrated that combined atorvastatin and metoprolol prophylaxis significantly reduced POAF incidence. In the intervention group, 8 patients (16.00%) developed POAF compared to 17 patients (34.00%) in the control group (*p* = 0.037, RR = 0.47, 95% CI: 0.23–0.96). This represents a 53% relative risk reduction and an absolute risk reduction of 18%, with a number needed to treat of 5.6 patients.

#### Secondary endpoints and clinical outcomes

Table 3 compares secondary outcomes between groups. The intervention demonstrated significant benefits across multiple clinically relevant endpoints: patients in the intervention group had significantly shorter hospital stay (7.37 ± 1.84 vs. 9.11 ± 2.46 days, *p* < 0.001), ICU stay (30.12 ± 7.47 vs. 35.26 ± 13.31 h, *p* = 0.002), and mechanical ventilation time (10.48 ± 4.72 vs. 15.55 ± 6.10 h, *p* < 0.001). The reduction in mechanical ventilation time likely reflects improved hemodynamic stability and reduced inflammatory response associated with the intervention, leading to faster respiratory recovery.

The incidence of major adverse cardiovascular events (MACE) was numerically lower in the intervention group (4.00% vs. 12.00%, *p* = 0.140), though this difference did not reach statistical significance, likely due to limited sample size and low event rates. Other secondary endpoints including 30-day readmission rate, postoperative bleeding, transfusion requirements, renal dysfunction, and mortality showed no significant differences, confirming the safety profile of the intervention.

#### Safety analysis

Table 4 presents adverse reaction data from the intervention group. Overall, 52.0% of patients reported at least one adverse reaction, but all were mild to moderate in severity, with no severe adverse reactions observed. The most common adverse reactions included muscle pain (10.0%), gastrointestinal discomfort (10.0%), fatigue (8.0%), and elevated transaminases (8.0%). No patients discontinued study medications due to adverse events, and all adverse reactions resolved without specific treatment or with symptomatic management.

**Table 4:** Incidence of drug Intervention-Related adverse reactions

Adverse Reaction	Mild [*n* (%)]	Moderate [*n* (%)]	Severe [*n* (%)]	Total [*n* (%)]
Muscle Pain	4 (8.0)	1 (2.0)	0 (0.0)	5 (10.0)
Elevated Transaminase	3 (6.0)	1 (2.0)	0 (0.0)	4 (8.0)
Gastrointestinal Discomfort	4 (8.0)	1 (2.0)	0 (0.0)	5 (10.0)
Dizziness	2 (4.0)	1 (2.0)	0 (0.0)	3 (6.0)
Fatigue	3 (6.0)	1 (2.0)	0 (0.0)	4 (8.0)
Bradycardia	2 (4.0)	1 (2.0)	0 (0.0)	3 (6.0)
Rash	2 (4.0)	0 (0.0)	0 (0.0)	2 (4.0)
Total	20 (40.0)	6 (12.0)	0 (0.0)	26 (52.0)

## Discussion

This study provides comprehensive evidence for POAF prediction and prevention in patients undergoing isolated aortic valve replacement, addressing a significant gap in current literature. Our findings identify age as a primary risk factor, with each year increase associated with a 12% higher odds of POAF. This strong age-related risk reflects the complex interplay of age-associated atrial structural and electrical remodeling. The pathophysiological basis includes increased atrial fibrosis through TGF-β pathway activation, enhanced oxidative stress from mitochondrial dysfunction, and autonomic nervous system imbalance [13–16].

The identification of stroke history as a powerful independent predictor (OR = 10.94) has important clinical implications for risk stratification. This association reflects the shared pathophysiology between cerebrovascular disease and atrial arrhythmogenesis, including chronic neuroinflammation, hypothalamic-pituitary-adrenal axis dysfunction, and persistent sympathetic hyperactivation [17–21]. Clinicians should consider patients with prior stroke as high-risk candidates requiring intensive POAF monitoring and preventive strategies [22, 23].

The inclusion of biomarkers (NT-proBNP and hs-CRP) in our prediction model represents a significant advancement in POAF risk assessment. Elevated NT-proBNP reflects increased atrial wall stress and activation of neurohormonal pathways that promote arrhythmogenesis through multiple mechanisms including stretch-activated channel activation, calcium homeostasis disruption, and RAAS activation [24–26]. Similarly, elevated hs-CRP indicates systemic inflammation that contributes to POAF through cytokine-mediated ion channel dysfunction, gap junction remodeling, and matrix metalloproteinase activation [27–30].

Our finding that prolonged operation time independently predicts POAF has direct implications for surgical planning and technique optimization. Each additional minute of surgery increases POAF risk by 2%, emphasizing the importance of efficient surgical techniques and the potential benefit of minimally invasive approaches [31–33].

The demonstrated efficacy of combined atorvastatin and metoprolol prophylaxis represents a significant advance in evidence-based POAF prevention for SAVR patients. The 53% relative risk reduction observed in our study aligns with previous research showing benefits of individual agents but demonstrates superior efficacy of combination therapy [34–37].

The mechanistic basis for this synergistic effect involves multiple complementary pathways. Atorvastatin’s pleiotropic effects include inhibition of HMG-CoA reductase and small G protein isoprenylation, leading to anti-inflammatory actions through NF-κB pathway suppression and PPARγ activation [36]. Additionally, atorvastatin enhances antioxidant capacity through eNOS upregulation while inhibiting NADPH oxidase activity [34]. Metoprolol contributes through β-adrenergic blockade, reducing sympathetic-mediated inflammation and providing cardioprotection through heart rate and myocardial oxygen consumption reduction [37]. The combination therapy appears to address multiple pathophysiological pathways simultaneously, explaining the superior efficacy compared to single-agent approaches.

The significant reduction in mechanical ventilation time observed with our intervention deserves particular attention. This benefit likely reflects improved hemodynamic stability through enhanced endothelial function, reduced inflammatory burden, and optimized autonomic balance. The anti-inflammatory effects of both medications may reduce pulmonary inflammation and improve gas exchange, while hemodynamic stability facilitates earlier extubation. This finding has important economic implications, as reduced ventilation time translates to decreased ICU costs and resource utilization.

Our simplified prediction model addresses a critical need for clinically practical risk assessment tools. Unlike existing models that often include numerous variables with limited clinical availability, our four-variable model (age, EuroSCORE II, NT-proBNP, hs-CRP) demonstrates excellent discriminative performance while maintaining clinical feasibility. The model’s excellent calibration and decision curve analysis results support its potential for clinical implementation, allowing clinicians to identify high-risk patients who would benefit most from intensive monitoring and preventive interventions.

The clinical utility of this model extends beyond risk prediction to inform treatment decisions. Patients identified as high-risk could be candidates for prophylactic therapy, enhanced monitoring protocols, or alternative surgical approaches. Furthermore, the model provides a framework for patient counseling and informed consent discussions regarding POAF risk.

This study’s mixed design provides both robust predictor identification and high-quality intervention evidence. The large retrospective cohort enables comprehensive risk factor analysis with adequate power, while the prospective RCT provides unbiased evidence for intervention efficacy. The focus on isolated aortic valve replacement addresses a specific gap in the literature, as most previous studies included mixed cardiac procedures with heterogeneous POAF risk profiles.

The development of a simplified, clinically practical prediction model represents a significant advancement over existing complex models. Our approach prioritizes clinical utility while maintaining excellent predictive performance, addressing the common limitation of academic models that are too complex for routine clinical use.

Several limitations warrant consideration in interpreting our results. The single-center design may limit generalizability across different institutions with varying surgical techniques, patient populations, and perioperative protocols. The relatively modest sample size of the RCT phase, while adequately powered for the primary endpoint, limits the ability to detect smaller differences in secondary endpoints and rare adverse events.

The absence of long-term follow-up represents an important limitation, as the impact of POAF prevention on long-term outcomes such as stroke, cognitive function, and quality of life remains unknown. Future studies should include extended follow-up to evaluate these clinically important endpoints.

External validation of our prediction model across multiple centers and diverse populations is essential before widespread clinical implementation. Such validation would confirm the model’s generalizability and identify any necessary calibration adjustments for different populations.

The lack of drug concentration monitoring limits our understanding of dose-response relationships and optimal dosing strategies. Future research should include pharmacokinetic studies to optimize dosing regimens and identify patients who may benefit from dose adjustments.

Future research should focus on several key areas to advance POAF prevention and management. Large-scale multicenter randomized trials are needed to confirm our findings and evaluate long-term clinical outcomes. Personalized medicine approaches incorporating genetic markers, advanced imaging parameters, and artificial intelligence algorithms may further improve risk prediction and treatment selection.

The development of real-time risk assessment tools using continuous monitoring data and machine learning algorithms represents an exciting frontier. Such tools could enable dynamic risk stratification and adaptive prevention strategies throughout the perioperative period.

Cost-effectiveness analyses are essential to inform healthcare policy and resource allocation decisions. While our intervention shows clinical benefits, formal economic evaluation is needed to demonstrate value in healthcare systems with limited resources.

Clinical Implications and Recommendations.

Based on our findings, we recommend that clinical teams consider implementing systematic POAF risk assessment using readily available clinical and biomarker data. High-risk patients identified through our prediction model should be considered for prophylactic therapy with combined atorvastatin and metoprolol, initiated 7 days preoperatively and continued until discharge.

The excellent safety profile observed in our study supports the routine use of this intervention in appropriate patients. However, individual patient factors including contraindications, drug interactions, and comorbidities should be carefully considered before treatment initiation.

Our findings support the development of institutional protocols for POAF prevention in SAVR patients, incorporating both risk assessment and evidence-based prophylactic strategies. Such protocols could standardize care and improve outcomes while reducing healthcare costs through shorter hospital stays and reduced complications.

This research contributes to the growing evidence base for personalized perioperative medicine, demonstrating that targeted interventions based on individual risk profiles can significantly improve patient outcomes. As the field moves toward precision medicine, our approach provides a practical framework for risk-stratified POAF prevention that can be implemented in current clinical practice while informing future research directions.

## Data Availability

No datasets were generated or analysed during the current study.
